# SNARE Modulators and SNARE Mimetic Peptides

**DOI:** 10.3390/biom12121779

**Published:** 2022-11-29

**Authors:** Mikhail Khvotchev, Mikhail Soloviev

**Affiliations:** 1 Department of Biochemistry, Center for Neuroscience, Faculty of Science, Mahidol University, Bangkok 10400, Thailand; 2 Department of Biological Sciences, Royal Holloway University of London, Egham, Surrey TW20 0EX, UK

**Keywords:** SNARE protein, SNARE motif, SNARE peptide, molecular self-assembly, SNARE mimetic, SNAREpins, membrane fusion, functional peptide, fusogen, clostridial neurotoxins

## Abstract

The soluble N-ethylmaleimide-sensitive factor (NSF) attachment protein (SNAP) receptor (SNARE) proteins play a central role in most forms of intracellular membrane trafficking, a key process that allows for membrane and biocargo shuffling between multiple compartments within the cell and extracellular environment. The structural organization of SNARE proteins is relatively simple, with several intrinsically disordered and folded elements (e.g., SNARE motif, N-terminal domain, transmembrane region) that interact with other SNAREs, SNARE-regulating proteins and biological membranes. In this review, we discuss recent advances in the development of functional peptides that can modify SNARE-binding interfaces and modulate SNARE function. The ability of the relatively short SNARE motif to assemble spontaneously into stable coiled coil tetrahelical bundles has inspired the development of reduced SNARE-mimetic systems that use peptides for biological membrane fusion and for making large supramolecular protein complexes. We evaluate two such systems, based on peptide-nucleic acids (PNAs) and coiled coil peptides. We also review how the self-assembly of SNARE motifs can be exploited to drive on-demand assembly of complex re-engineered polypeptides.

## 1. Introduction

### 1.1. Overview of Intracellular Membrane Fusion

Eukaryotic cells critically depend on the vesicular trafficking of biomolecules. Membrane-bound organelles of various sizes and shapes originate from intracellular donor compartments and fuse with target compartments in a spatially and temporally regulated manner tailored to the specific requirements of the locale. Vesicular trafficking between ER and Golgi compartments appears to be a continuous and steady process subtly regulated by several mechanisms. In sharp contrast, the fusion of most synaptic vesicles with the plasma membrane at brain synapses is triggered by an influx of calcium with temporal resolution in the microsecond range [1]. Precise molecular events leading to membrane fusion remain enigmatic but likely involve bringing lipid bilayers in close opposition and disrupting local lipid organization [2]. Most intracellular membrane fusion is administered by members of several evolutionarily conserved protein families with the soluble N-ethylmaleimide-sensitive factor (NSF) attachment protein (SNAP) receptor (SNARE) proteins having a central role [3]. SNARE proteins are present on both donor (transport vesicle) and acceptor (target organelle) membranes and are often classified into v-SNAREs and t-SNAREs, respectively.

### 1.2. Native SNARE Protein Structure and Function

**Figure 1:**
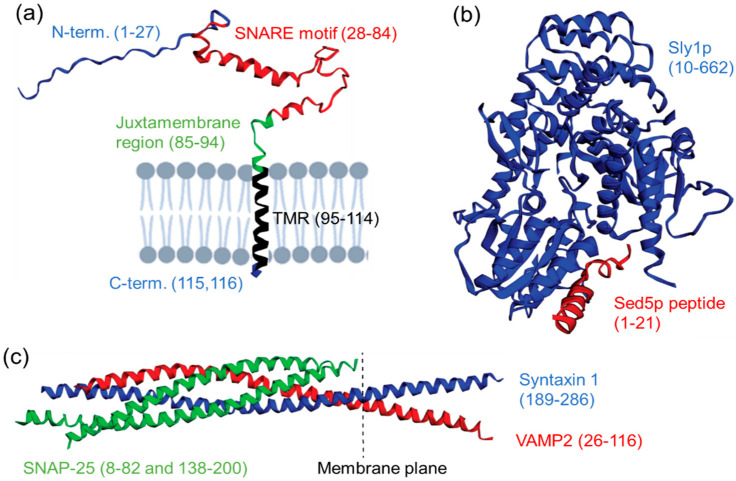
Molecular anatomy of SNARE proteins and their complexes. (**a**) Model of rat VAMP2 inserted into lipid bilayer. Amino acid residues indicate boundaries of VAMP2 structural regions (TMR denotes transmembrane region). PDB accession#: 2KOG. (**b**) Structure of an N-terminal peptide of yeast SNARE protein Sed5p (red) in a complex with yeast SM protein Sly1p (blue). PDB accession#: 1MQS. (**c**) Structure of the rat neuronal SNARE complex. PDB accession#: 3HD7.

The archetypical SNARE protein VAMP2, also known as synaptobrevin 2, is a small intrinsically disordered single pass membrane protein. The short unstructured N-terminal sequence (usually 10–30 residues long) has no apparent function in many SNAREs. In syntaxins, the N-terminal peptide forms a binding interface with members of the conserved SM protein family (Sec1/Munc18 proteins), also essential for membrane fusion [4]. The central part of the SNARE protein is occupied by a heptad repeat sequence called a SNARE motif, which is 60–70 residues long (Figure 1). Four separate SNARE motifs assemble spontaneously into a highly stable intertwined α-helical bundle called a SNARE complex. SNARE complex assembly proceeds slowly in vitro but is highly accelerated by SNARE-regulating proteins in vivo [5]. Each SNARE protein typically contributes a single SNARE motif but some SNARE proteins (e.g., SNAP-25) contain two motifs, both of which can participate in a single SNARE complex. All known functional SNARE complexes are formed by three or four individual SNARE proteins with at least one SNARE protein present on each membrane (trans-SNARE complex). Following membrane fusion, SNARE proteins engaged in a SNARE complex are found on a single membrane (cis-SNARE complex) and are disassembled for the next round of membrane fusion by AAA ATPase NSF in an energy-dependent process [6]. SNARE-complex formation occurs by a zippering mechanism, forcing two participating membranes into close opposition. In vitro, both parallel (N→C) and antiparallel (N→C/C→N) configurations of SNARE motifs within a SNARE complex exist; however, in vivo only parallel assemblies result in functional SNARE complexes [7]. At present, it is not clear how many SNARE complexes are required to fuse the membranes—any number of complexes from one to several have been reported previously [8,9]. SNARE complexes form with some degree of promiscuity, indicating that the specificity of intracellular membrane fusion depends on additional mechanisms. Non-productive or not fully assembled SNARE complexes were also identified, with their functional significance subject to debate.

The C-terminal part of a typical SNARE protein contains a short juxtamembrane linker sequence (~10 residues), transmembrane region (~20 residues) and a few C-terminal residues found on the opposite side of the membrane (intralumenal or extracellular). The juxtamembrane region in many SNAREs contains conserved basic and bulky hydrophobic residues and appears to have membrane-active properties important for catalyzing membrane fusion [10,11]. The transmembrane region not only anchors SNARE proteins to a membrane but likely plays additional roles in SNARE multimerization, the regulation of membrane fusion and fusion pore dynamics [12,13,14]. C-terminal localization of the transmembrane region in SNARE proteins necessitates the use of specialized translocation complex Get1/2 for posttranslational membrane insertion [15]. A subset of SNARE proteins (e.g., SNAP-25) lack transmembrane regions and are attached to a membrane by hydrophobic posttranslational modifications (palmitoylation). Several studies have indicated that the mode of membrane attachment is essential for SNARE function [16] while others found them interchangeable [17,18]. Important structural variation found the presence of an autonomously folded N-terminal domain in several SNAREs that regulates their sorting and the availability of the SNARE motif for SNARE-complex assembly [19,20]. These SNAREs transition between ‘open’ (available for SNARE complex formation) and ‘closed’ conformations under tight regulatory control by SM proteins and other factors [21,22]. In summary, SNARE proteins operate via multiple protein–protein and protein–lipid binding interfaces. Critical interfaces are formed by SNARE motifs in the SNARE complex, structurally diverse SNARE–SM protein interfaces and SNARE–lipid interfaces involved in membrane attachment and lipid regulation. In addition, SNAREs interact with several regulating and chaperoning proteins (e.g., α-synuclein, NSF/α-SNAP complex). Specialized interfaces are found in SNAREs involved in calcium-triggered membrane fusion [23]. Multiple studies implied that SNARE proteins are involved in cellular functions not directly related to membrane fusion. The SNARE binding interface was identified in multiple ion channels and transporters with implications for the direct regulation of their function by SNAREs [24,25]. Modifying or manipulating functional interfaces in SNARE proteins is a powerful tool to dissect their cellular roles and to modulate their activity.

### 1.3. Molecular Manipulations of SNARE Proteins

SNARE proteins are involved in virtually all key cellular processes and serve as a powerful tool to tease out relevant molecular mechanisms. In addition, multiple pathological processes and human diseases are directly or indirectly related to SNARE function. These include SNAREopathies [26], diseases of nervous, endocrine, and immune systems [27], neurodegenerative diseases [28] and developmental disorders [29]. The important roles of SNARE proteins in normal and pathophysiological processes are exemplified by clostridial neurotoxins. Studies on clostridial neurotoxins uncovered the essential function of neuronal SNAREs in the fusion of synaptic vesicles with the plasma membrane (reviewed in [30]). Light chains of the toxins cleave neuronal SNARE proteins at specific sites, releasing various fragments of VAMP2, SNAP-25 and syntaxin 1, and inactivate membrane fusion at the affected sites. The inhibition lasts for several months in some cases, turning botulinum toxins into biologicals widely used to treat a growing list of medical and cosmetic conditions. The high specificity of clostridial neurotoxins creates obstacles for their applications. While targeted cellular delivery of the toxins can be modified by protein engineering [31], it remains challenging to expand the SNARE repertoire cleaved by them. The development of small molecules that target SNAREs is of great importance but few have been identified so far. Several plant extracts containing polyphenols inhibit SNARE-complex formation [32]. In high concentrations, a polyphenolic substance, myricetin, arrests SNARE-complex formation by intercalating into assembling SNARE intermediates [33,34,35]. Genetic and biochemical studies provided evidence that general anesthetics may bind [36] and modulate individual SNAREs and SNARE complexes in *C. elegans* [37] and mammalian neurons [38]. Specific classes of lipids (phosphatidylinositides, arachidonic acid, sphingomyelin) were shown to regulate SNAREs and SNARE-complex assembly [39,40,41]. SNARE functional interfaces are often large and extended, making it difficult for small molecules to modify them significantly. Peptides are better poised to fulfill this role, particularly with current advances in peptide chemistry and intracellular delivery. Peptides also emerged as an attractive tool to mimic SNARE function to study the fundamental mechanism of membrane fusion and for various biomedical and bioengineering applications [42]. 

## 2. Peptides Modifying SNARE-Protein Functional Interfaces

### 2.1. SNARE Complex

**Figure 2:**
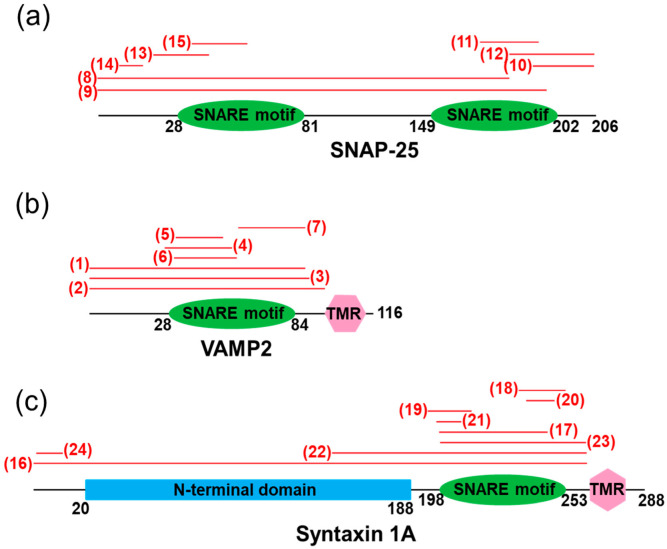
Mapping of the known SNARE-derived functional peptides. Neuronal SNARE proteins, (**a**) SNAP-25, (**b**) VAMP2, and (**c**) syntaxin 1A are schematically shown with N-terminal domain, SNARE motifs and transmembrane region (TMR) indicated. Numbering of amino acid residues is shown below in black for rat SNARE proteins. Functional peptides are mapped by red bars. The numbering (in red) corresponds to the peptide entry number in the Supplementary Table S1.

Upon cleavage by clostridial neurotoxins, VAMP2 and syntaxin 1, larger fragments containing most of the SNARE motif are released from the membrane, whereas a large fragment of SNAP-25 remains membrane-bound. SNARE cleavage products do not support membrane fusion and exert a dominant negative effect by forming non-productive SNARE complexes with intact SNARE proteins. Injection or viral delivery of SNAP-25 fragments into Aplysia cholinergic neurons, where such fragments resembled cleavage products generated by botulinum neurotoxins BoNT A and E (residues 1–180 and 1–197), reduced postsynaptic currents by 50% and inhibited glutamate release in cultured hippocampal neurons by 40–60% [43]. Well over 20 such functional peptides have been reported to date (Figure 2). Their sequences are further detailed in the Supplementary Table S1, and a few notable examples are detailed below. 

When transfected into insulinoma cells, a SNAP-25 fragment (residues 1–197) inhibited insulin secretion evoked by depolarization or high glucose to levels comparable to BoNT A application [44]. Notably, in permeabilized PC12 cells treated with BoNT E, calcium-dependent norepinephrine release is partially rescued by adding a 26-mer peptide corresponding to the cleaved C-terminal coil of SNAP-25 [45]. Rescue can be enhanced by the introduction of a D186A mutation into the peptide sequence. This result indicates that the continuity of the C-terminal helix is not essential for SNAP-25 function. However, a similar approach applied to VAMP2 cleaved by BoNT D failed to rescue exocytosis. A cytoplasmic fragment of VAMP2 released by the tetanus toxin (residues 1–93) inhibited neurotransmitter release at an *Aplysia* cholinergic synapse [46]. A similar fragment of VAMP2 (residues 1–96) inhibited fusion-pore expansion and blocked the release of neurotransmitters and peptides from transfected astrocytes [47]. VAMP2 (residues 1–94) and syntaxin 1 fragments (residues 1–265) have been used extensively to block lipid and content mixing in liposome-based reconstitution assays [48]. Notably, the treatment of mouse N2A and human SH-SY5Y neuroblastoma cell lines but not of neurons with BoNT C or BoNT C/D, targeting syntaxin 1 and VAMP2, resulted in significant cytotoxicity [49].

Cytotoxicity was also observed with syntaxin 1 (201–245) and VAMP2 (25–52) peptides, which can form aberrant SNARE complexes. A similar approach was applied to SNARE proteins that are not natural substrates for clostridial neurotoxins. A SNAP-23 fragment lacking 8 C-terminal amino acids (modeled after SNAP-25 cleaved by BoNT E) inhibited constitutive exocytosis of glutamate transporter EAAC1 in transfected C6 glioma cells [50]. SNAP-25 cleavage by BoNT A, C and E releases short C-terminal peptides containing the C-terminal part of the second SNARE motif. Multiple studies indicated that these peptides inhibit membrane fusion mediated by SNAP-25. A 20-mer peptide encompassing the C-terminal region of SNAP-25 (residues 187–206) blocked calcium-evoked catecholamine release from permeabilized chromaffin cells with IC_50_ ~20 μM [51]. A 26-mer peptide modeled after SNAP-25 cleavage by BONT E also inhibited exocytosis in permeabilized chromaffin cells [52]. Finally, SNAP-25 C-terminal peptides (residues 187–206, 170–189 and 181–206) inhibited neurotransmission in an *Aplysia* cholinergic synapse by ~40% after injection into the presynaptic neuron [53].

Several peptides derived from the SNARE motif sequence of various SNARE proteins have been demonstrated to inhibit SNARE-dependent membrane fusion. Peptides derived from the N-terminal region of SNAP-25 inhibited SNARE-complex formation and exocytosis from permeabilized chromaffin cells. Highly potent peptide containing residues 22–44 also protected primary hippocampal neurons against glutamate-induced neurotoxicity and disrupted the SNAP-25/syntaxin 1 binary complex [54]. The hexapeptide Ac-EEMQRR-NH2 (called argireline) patterned after the N-terminal part of the first SNAP-25 SNARE motif (residues 12–17) interfered with the assembly of the SNARE complex, and inhibited calcium-dependent catecholamine release from permeabilized chromaffin cells [55]. Argireline penetrated through the skin epidermal layer and displayed anti-wrinkle activity in human subjects. Syntaxin 1 SNARE motif peptides (residues 229–251 and 197–219) inhibited calcium-induced insulin secretion from permeabilized pancreatic beta-cells, increased basal insulin release and had no effect on insulin release induced by non-hydrolysable GTP analogs [56]. Similar myristoylated peptides (residues 233–245 and 200–212) inhibited glucose-induced insulin secretion from intact beta-cells [57]. The introduction of a longer syntaxin 1 peptide containing the entire SNARE motif (residues 162–265) by a patch electrode inhibited neurotransmitter release in primary hippocampal neurons [58].

Most peptides cannot diffuse efficiently through biological membranes. To improve intracellular delivery, SNARE-derived peptides were fused with protein-transduction sequences (e.g., TAT sequence). TAT-fused syntaxin 1 peptide (residues 202–265) was effectively taken up by intact cells and inhibited regulated exocytosis in pancreatic beta-cells [59] and PC12 cells [60]. Furthermore, 17-mer peptides derived from the N-terminal region of SNARE motif in VAMP2 and VAMP8 inhibited SNARE-complex formation and reduced mast-cell degranulation [61]. When fused with protein-transduction sequences, these peptides improved the symptoms of atopic dermatitis in mouse models.

SNARE motif peptides were also used to test the parallel zippering model of SNARE-protein function. This model predicts that the zippering is initiated at the N-terminus of the SNARE motif and proceeds towards the C-terminus. Therefore, N-terminal peptides are predicted to inhibit membrane fusion more effectively than C-terminal peptides, by arresting the nucleation of the SNARE complex. In liposome-fusion assays with reconstituted neuronal SNARE proteins, the N-terminal VAMP2 peptide (residues 29–56) potently blocked membrane fusion [62]. In contrast, the C-terminal peptide (residues 5792) accelerated membrane fusion, likely by structuring the membrane-proximal coiled coil region in the t-SNARE.

A search for peptide inhibitors of SNARE complex formation and membrane fusion was conducted using the combinatorial 17-mer α-helix-constrained peptide library [63]. A potent hit was identified, with amino acid sequence: acetyl-SAAEAFAKLYAEAFAKG-NH2, unrelated to SNARE-protein sequences. The peptide blocked calcium-evoked catecholamine secretion from permeabilized chromaffin cells and glutamate release from primary hippocampal neurons.

In summary, functional peptides derived from the SNARE motif sequence potently inhibit membrane fusion by forming non-productive SNARE complexes with endogenous SNAREs. These peptides are active in a wide range of functional assays and systems from in vitro reconstituted liposome-fusion assays to permeabilized and intact cell-based assays. When fused with protein-transduction sequences, the peptides can efficiently penetrate through the biological membranes, opening a path to their use in basic and applied science.

### 2.2. SNAREs/SM Proteins

The important role of SNARE regulators and their role in the process of intracellular membrane fusion, mediated by SNARE proteins, has been recognized long ago, e.g., [64]. In vivo, SNARE proteins are critically assisted by SM proteins, an evolutionarily conserved protein family essential for intracellular membrane fusion. SM proteins have been shown to interact with individual SNAREs (v-SNAREs, syntaxins) and SNARE complexes by structurally diverse mechanisms [65,66]. SM protein Munc18-1 binds the N-terminal domain of syntaxin 1 in the closed conformation (the domain is folded back onto the SNARE motif, preventing SNARE complex assembly) and, independently, to the N-terminal sequence of syntaxin 1 (~20 N-terminal residues). Several lines of evidence suggest that SM proteins guide and chaperone SNARE proteins to facilitate SNARE-complex assembly, but conflicting data exist on the functional significance of diverse SM/SNARE binding modes. For example, the binding of SM protein Munc-18 to syntaxin 1 N-terminal peptide was shown to be essential for neurotransmission in *C. elegans* [67]. Other studies indicated that this binding mode is not required but plays a more subtle regulatory role in *C. elegans*, PC12 cells [68] and mammalian neurons [69]. It remains challenging to use peptides for the analysis of SM/closed syntaxin complexes, because the binding interface is large and convoluted, whereas the binding affinity is low nanomolar or higher. In contrast, the interaction via the short N-terminal sequence of syntaxins is ideally suited for this approach. N-terminal syntaxin 1A peptide (residues 2–16) interfered with Munc18-1/neuronal SNARE-complex assembly and inhibited neurotransmission at the calyx of Held synapse [70]. Peptide containing the D3R mutation, which disrupts the interaction, had no effect.

Notably, cell-based secretion assays indicated that syntaxin fragments may inhibit membrane fusion by acting as a sink for endogenous SM proteins and not by forming aberrant SNARE complexes [70,71]. SM proteins Munc18-1 and 3 contain a SNARE-like peptide (residues 327–351) that structurally and functionally resembles the C-terminal peptide of VAMP2 (residues 60–84) [72]. Both peptides activated membrane fusion by SNAREpins in reconstituted liposome-based assays by a similar mechanism.

### 2.3. Other Functional Interfaces in SNARE Proteins Involved in Membrane Fusion

SNARE proteins are thought to initiate membrane fusion by bringing two membranes in close opposition. Several studies have indicated that juxtamembrane and transmembrane regions in SNAREs are required to disrupt fusing membranes and to catalyze lipid mixing. Juxtamembrane peptide from VAMP2 (residues 79–94) was required for membrane fusion in reconstituted liposome-based assays but could be functionally substituted with an unrelated membrane-destabilizing peptide [73]. Mutations in conserved hydrophobic or positively charged amino acid residues or extending the length of the juxtamembrane linker region by at least two residues inactivated SNARE function. Vicinal tryptophan residues in the juxtamembrane region are highly conserved in most VAMP2 proteins. In vitro studies with peptides derived from the juxtamembrane and transmembrane regions of Drosophila VAMP2 (residues 75–121) showed that peptides containing two, one or no tryptophans were positioned differently in the micelle, indicating that these residues are important for VAMP2 orientation in the vesicle membrane [74]. The VAMP2 peptide containing both regions (residues 83–116) enhanced stalk and pore formation in highly curved vesicles in the presence of phosphatidylserine [75].

In vivo, SNARE complexes are disassembled by AAA ATPase NSF and accessory proteins. Inhibiting NSF function should increase non-productive cis-SNARE complexes and block membrane fusion. TAT sequence-fused 22-mer peptides were derived from NSF-inhibited exocytosis of the von Willebrand factor in endothelial cells [76]. Peptides targeted different domains of NSF responsible for various functions. The most potent peptide was obtained from the sequence involved in NSF homooligomerization. 

Multiple proteins regulate SNARE function, including the abundant presynaptic protein α-synuclein, linked to Parkinson’s disease pathology. α-synuclein was shown to bind VAMP2 and to promote SNARE-complex assembly in vitro and in vivo [77]. α-synuclein forms oligomers that also bind VAMP2, cluster small vesicles and inhibit membrane fusion [78]. These effects were reversed by 30-mer peptides derived from the α-synuclein C-terminal region involved in VAMP2 interaction, with the most potent peptide containing residues 96–125.

Calcium-triggered membrane fusion is of critical importance for nervous, endocrine, and immune systems. SNARE proteins are not intrinsically sensitive to calcium; therefore, a calcium sensor protein is incorporated into the SNARE machinery [79]. The best studied calcium sensors are a family of double C2 domain membrane proteins called synaptotagmins. Synaptotagmin 1 C2B domain forms a primary interface with the partially assembled SNARE complex and enables calcium regulation [80].

Recent studies identified potent functional peptides that disrupt the primary interface and block calcium-triggered membrane fusion [81,82]. Because the primary interface is linearly extended, internally crosslinked (stapled) peptides were designed and tested in reconstituted liposome-based assays. The most effective peptide was derived from SNAP-25 (residues 37–53) and contained four nonnatural amino acids for stapling. Remarkably, this peptide potently blocked the stimulated secretion of mucin in human or mouse airway epithelium cells. Calcium-dependent secretion of mucin is mediated by a different set of proteins (SNAREs: VAMP8, syntaxin 3 and SNAP-23; calcium sensor: synaptotagmin 2); however, the primary interface is sufficiently conserved in both neuronal and airway cells. To allow efficient intracellular delivery, the stapled peptide was conjugated with a protein-transduction sequence (PEN or TAT). When applied in aerosol form, the peptide markedly reduced the accumulation of mucin in mouse airways, which has therapeutical implications for the treatment of many lung diseases.

### 2.4. SNAREs/ion Channels and Transporters

The interaction of syntaxin 1 with N-type Ca^2+^-channels was initially demonstrated by co-immunoprecipitation studies [83,84] leading to a simple model of how calcium entry and fast neurotransmission can be coupled at the synapse. The interaction was mapped to the cytoplasmic loop II-III in N-type but not Q- or L-type Ca^2+^-channels [85,86]. The 87-mer peptide derived from the loop sequence, termed the synprint, was shown to block the interaction of the channel with the C-terminal region of syntaxin 1 (residues 181–288). The synprint peptide also interacted with syntaxin 1 and SNAP-25 only in the presence of calcium with EC50 ~ 20 μM. Synprint interaction with syntaxin 1 and SNAP-25 displayed distinct calcium dependence for various P/Q-type Ca^2+^-channel isoforms [87]. The introduction of synprint peptides into presynaptic cervical ganglion neurons modified neurotransmission by inhibiting fast synaptic responses and augmenting slow asynchronous synaptic responses and paired-pulse facilitation but had no effect on calcium currents [88]. Peptides derived from L-type Ca^2+^-channels had no effect. The introduction of synprint peptides into Xenopus embryos decreased the calcium sensitivity of neurotransmitter release in spinal neurons [89]. In another study, mutation in the P/Q-type Ca^2+^-channel (localized in loop I-II) prevented the modulation of channel properties by syntaxin 1 and SNAP-25 in an HEK293 cell-based overexpression model [90]. 

In addition, voltage-gated K+-channels Kv2.1 have been shown to interact functionally and structurally with neuronal SNARE proteins. Syntaxin 1 inhibited currents of the Kv2.1 channel overexpressed in HEK293 cells by interacting with the C-terminus of the channel [91]. The C-terminal peptide blocked the interaction and relieved channel inhibition, whereas the N-terminal peptide had no effect. Peptides derived from the C-terminal region of Kv2.1 channels disrupted interaction with syntaxin 1 and inhibited regulated exocytosis in cracked PC12 cells [92]. The injection of Kv2.1 C-terminal peptides into oocytes overexpressing Kv2.1 channels and syntaxin 1 reversed the effect of syntaxin 1 on the activation/inactivation of the Kv2.1 channel [93]. Surprisingly, SNAP-25 also inhibited the Kv2.1 current in pancreatic beta-cells by interacting with the N-terminus of the channel [94]. This effect can be reversed by the introduction of the N-terminal Kv2.1 peptide, which disrupts the interaction with SNAP-25, but not by the C-terminal peptide. A fragment of SNAP-25 (residues 1–180) inhibited the Kv2.1 current and enhanced glucose-dependent insulin secretion from pancreatic beta-cells [95]. The SNARE motif of syntaxin 1 inhibited the sodium current of ENaC in Xenopus oocytes and interacted with the C-terminus of ENaC[96]. 

The list of membrane proteins modulated by SNAREs includes several dozens of ion channels and neurotransmitter transporters, raising significant concerns about the general validity of these observations. SNARE proteins and particularly syntaxin 1 have been shown to interact promiscuously with multiple proteins in pulldown and co-immunoprecipitation assays [97]. Functional studies have often relied on the heterologous overexpression of membrane proteins (ion channels, transporters, SNAREs) in mammalian cell lines and oocytes. The overexpression of these proteins, in particular neuronal SNAREs, has profound effects on intracellular trafficking in many cases [70,71]. No specific sequence motif has emerged as a SNARE binding site, with multiple SNAREs binding to different regions of the same channel protein. The initial hypothesis of the direct coupling of SNARE fusion machinery to calcium channels at the presynapse was refuted by recent studies of active zone proteins involved in channel clustering [98].

## 3. SNARE-Mimetics, Functional Peptides Mediating Membrane Fusion

### 3.1. Overview of SNARE-Mimicry-based Fusogenic Systems

Intracellular membrane fusion is a complex process that involves multiple proteins, lipids, and other factors. The development of simplified systems that allow for specific and efficient membrane fusion in the physiological environment would greatly benefit a wide range of fields, from fundamental studies of cellular function and membrane fusion to biomedical and bioengineering applications. Ideally, (i) they should use a limited number of components (optimally, two found on each of the fusing membranes), and (ii) fusogens should be small and simple molecules. Many viruses use binary fusogenic systems (cellular receptor-viral fusogen); however, the proteins involved are large and multi-subunit assemblies [99].

An important step towards the design of SNARE-mimetics was the demonstration that SNARE proteins themselves (SNAREpins) can mediate membrane fusion when reconstituted in proteoliposomes [48] or the fusion of cells in ‘flipped out’ configuration [100]. Higher rates of membrane fusion were observed when all regulatory sequences were stripped from the SNAREpins leaving only SNARE motifs attached to the membrane [101]. However, SNAREpins have several disadvantages: (i) membrane fusion mediated by them is slow; (ii) at least three SNAREpins are required; and (iii) while significantly smaller than viral fusogens, they contain ~ 100 amino acid residues at minimum.

Recent studies have uncovered naturally occurring SNARE mimicry in intracellular bacterial pathogens that hijack endocytic pathways to grow in induced vacuoles (reviewed in [102]). Bacteria use SNARE mimicry to facilitate some fusion events (vesicular delivery to infected vacuoles) and inhibit others (fusion of endosomes/vacuoles containing bacteria with lysosomes). Legionella LegC proteins mimic t-SNAREs and form a SNARE-like complex with VAMP4 (substituting syntaxin 6) that cannot be disassembled by NSF [103,104]. This results in membrane fusion, recruiting VAMP4 vesicles to deliver membranes and other biomolecules needed for Legionella growth and multiplication in vacuoles. Chlamydia IncA protein also mimics SNAREs to enable bacteria replication in vacuoles [105]. Proteins that form aberrant complexes with endogenous SNAREs and inhibit membrane fusion were identified in Legionella and Chlamydia [106]. These findings raise the intriguing possibility that SNARE-mimetics can be designed to work with endogenous SNARE proteins in hybrid membrane fusion reactions.

Several approaches to mimic intracellular membrane fusion have been developed, including the use of small molecules (cis-diols/boronic acid), vancomycin/D-ala dipeptide, hydrogen bonding in lipidated small molecules (cyanuric acid/melamine), double-stranded DNA structures, hybrid peptide-nucleic acid (PNA) modules and coiled coil peptides (reviewed in [42]). In many cases, they fail to recapitulate properties of the native membrane fusion completely. Artificial fusogens often mediate efficient lipid but not content mixing, are not inhibited by inverted cone-shaped lipids and are sensitive to membrane-curvature stress. The vancomycin glycopeptide/D-Ala dipeptide system, which requires a membrane disruptor (e.g., magainin II antimicrobial 23-mer peptide) for efficient membrane fusion, is inherently leaky [107]. Here, we focus on the most promising and highly developed modular SNARE-mimetics to date: peptide-nucleic acids and coiled coil peptides.

### 3.2. Peptide-nucleic acid (PNA) Fusogens

**Figure 3:**
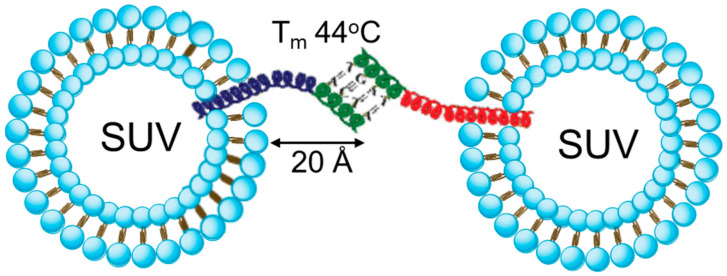
SNARE mimicry by peptide-nucleic acid (PNA) fusogens. PNA pairs were inserted into artificial liposomes via native transmembrane regions of SNARE proteins (modified from [108]). SUV—small unilamellar vesicle. Optimal length of the PNA linker for membrane fusion is shown in angstroms. Tm of DNA duplex is indicated. Antiparallel orientation is shown.

Several β-PNA pairs were designed and used in artificial liposome fusion assays [108]. PNAs contained native transmembrane regions derived from neuronal SNAREs, VAMP2 and syntaxin 1, and a rigid peptide linker with four DNA bases attached to allow DNA double strand formation (T_m_ ~44°C). This design permits only the antiparallel orientation of the PNA complexes (Figure 3) in contrast to SNARE-mediated membrane fusion, which requires parallel orientation and the zippering of the SNARE motifs. Liposomes with reconstituted β-PNA pairs displayed efficient lipid and content mixing at elevated temperatures (optimal at 35–45°C and inhibited at 55°C) with moderate content leak. Full fusion occurred in PNA pairs with a short extramembrane part (~20 Å). When the length was doubled, only hemifusion was detected. Additional PNA pairs were designed to test the effect of transmembrane regions and the recognition-motif orientation [109,110]. In these studies, lipid mixing was improved in PNAs with parallel orientation of recognition motifs and when transmembrane regions were derived from v- and t-SNARE pairs. Interestingly, peptides derived from transmembrane regions of VAMP2 and syntaxin 1 and low complexity hydrophobic model sequence LV-peptides can drive liposome fusion, particularly in the presence of liposome-aggregating agents [111,112].

### 3.3. Coiled coil Peptides, True SNARE-Mimetics

To date, the best characterized reduced SNARE-mimic system is based on a pair of positively and negative charged peptides that can form stable coiled coil complex with K_a_ ~10^−7^ M [113]. Cationic peptide K, (KIAALKE)x3, and anionic peptide E, (EIAALEK)x3 contain three heptad repeats and are ~ 1/9 the size of a small SNARE protein such as VAMP2 containing eight heptad-repeat SNARE motif [114]. The peptides include a flexible PEG linker and were anchored to the membrane by lipidation. When reconstituted in artificial liposomes, E/K peptides induced efficient lipid and content mixing without significant leakage. Importantly, E/K peptides were fusogenic when inserted in parallel or antiparallel orientation, indicating that the standard model of SNARE-protein zippering may not apply to these SNARE-mimetics [115]. A different study found that the parallel orientation of coiled coils results in lipid mixing, whereas the antiparallel orientation leads to liposome docking only [116]. Unfortunately, E/K peptides used in that study contained a glycine linker, rendering it difficult to directly compare the results of both studies. 

Detailed biophysical studies of the E/K peptide system indicated that two peptides have significantly different properties in the membrane environment [117]. Peptide E does not interact with the membrane and remains fully available for coiled coil assembly with the counterpart peptide. In contrast, peptide K binds and shallowly penetrates the membrane and exists in an equilibrium of membrane-bound and coiled coil assembly-ready states [118]. Subsequently, peptide K appears to have a dual role in membrane fusion: (i) similar to the SNARE motif in SNARE proteins, peptide K brings two membranes into close opposition by forming a coiled coil assembly with peptide E; and (ii) similar to the juxtamembrane region conserved in many SNARE proteins, peptide K destabilizes membranes to initiate membrane fusion. A recent study used molecular simulations to analyze E/K peptide interaction with membranes [119] and arrived at different conclusions. Both peptides were repulsed or attracted by membranes depending on the specific lipid composition.

**Figure 4:**
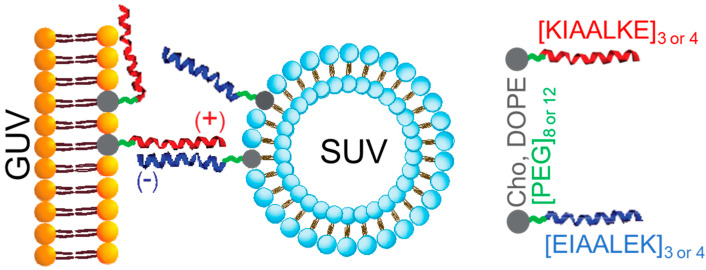
SNARE mimicry by E/K coiled coil peptides. E (blue coil) and K (red coil) peptides inserted into artificial membranes by a covalently linked lipid moiety. One peptide is positively charged (+) and additionally interacts with membrane, another peptide is negatively charged (-). Antiparallel orientation is shown. GUV—giant unilamellar vesicle. SUV—small unilamellar vesicle. Each peptide is lipidated (in grey: Cho—cholesterol, DOPE—1,2-dioleoyl-sn-glycero-3-phosphatidylethanolamine) and contains a PEG linker of variable length (in green). Sequence of a basic (in red) and an acidic (in blue) heptad repeat is shown. Three or four repeats are included for optimal performance.

The E/K system is highly flexible and adaptable and has been extensively optimized for various applications. By changing parameters, such as the lipid:E/K peptide ratio, membrane fusion can be controlled to enable two liposomes to fuse or to facilitate multiple rounds of fusion resulting in a single giant liposome [120]. Increasing the stability of coiled coil assembly by increasing the number of heptad repeats in E/K peptides facilitated the rate of membrane fusion [121]. The mode of membrane attachment can also be varied. E/K peptides fused with native transmembrane regions of SNARE proteins facilitate lipid and content mixing in liposome-fusion assays [122]. However, the optimal mode of membrane attachment is by lipidation through a cholesterol moiety [123]. A flexible PEG spacer length is also important and varies. Two peptides, PEG_12_ E and PEG_8_ K (Figure 4), gave optimal results. Peptide stapling using ortho-xylene as a crosslinker in the K peptide increased coiled coil stability and fusogenicity but had no effect on the interaction of the K peptide with the membranes [124].

Naive membranes can be modified by the application of optimized E/K peptides to increase their fusogenicity [125]. The E/K system was used to facilitate the fusion of giant unilamellar vesicles (GUVs) and large unilamellar vesicles (LUVs) under physiological conditions. While efficient lipid mixing was observed, the content mixing was poor due to LUV clustering on the surface of GUV target membranes [126]. The non-ionic detergent Tween 20 improved content-mixing between GUVs and LUVs, likely by softening the target membrane. Thus, E/K peptides have the potential to be employed for drug delivery in biological systems as evidenced by the efficient targeted delivery of the small molecule anionionophore into GUVs loaded into LUVs [127]. The E/K system can be further extended by the development of a small library of lipidated coiled-coil-forming orthogonal peptides, with implications for membrane fusion specificity [128].

## 4. SNARE-Inspired Polypeptide-based Binary Protein Assembly Systems

**Figure 5:**
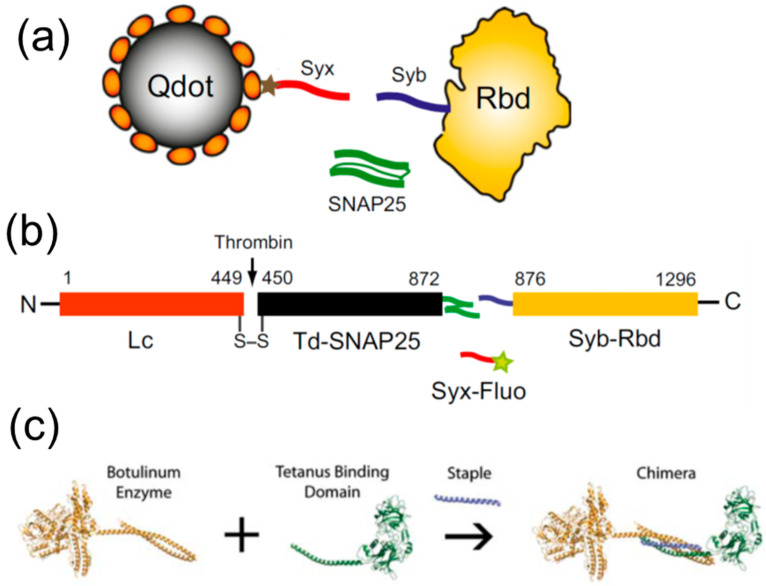
SNARE tagging. (**a**) On-demand linking of surface-derivatised quantum dots to the receptor-binding domain of botulinum neurotoxin (Rbd) using the SNARE domain of a native SNAP-25 protein as a ‘staple’. (**b**) Self-assembly linking of SNARE-tagged botulinum neurotoxin domains into a functional toxin using SNARE domain of native syntaxin protein as a ‘staple’; (**a**) and (**b**) reproduced with permission from [133]. (**c**) Stepwise assembly of an artificial clostridial chimeric protein built from botulinum type A protease/translocation domain from Clostridium botulinum (devoid of motor-neuron-targeting domain) and the tetanus toxin binding domain from Clostridium tetani (capable of targeting central neurons, but not motoneurons). A SNARE domain of native syntaxin protein is used as a ‘staple’. Reproduced under CC-BY-4.0 license from [134].

The coiled coil SNARE domain has also been explored in a variety of protein engineering applicators. The relative simplicity of the coiled coil protein assembly principle and the unique ability of three protein constituents of a SNARE complex to assemble in a sequence-dependent manner (unlike many other simple coiled coils widely explored to date) has inspired a variety of SNARE-mimicking molecular designs. Over the last few decades, many seminal publications have scrupulously detailed the principles and the kinetics of self-assembly of the ternary native SNARE complex [129,130,131,132]. More recently, the SNARE self-assembly principle was used to create a SNARE-inspired system to drive the molecular assembly of entirely different proteins, using SNARE polypeptides as self-assembling tags. The early designs relied on full length SNAP-25 as a large protein ‘staple’ to link two tagged proteins (Figure 5) [133]. Such protein assembly relies on the natural SNARE helix’s topology and uses the SNAP-25 double helix to play the role of a connecting staple. Whilst such approach requires only relatively short tags to be added to the protein being connected, the ‘staple’ itself is relatively large, being based on the two SNAP-25 helixes. The alternative assembly with only a single alpha helix being a ‘staple’ has also been proven to work and two new functional multinodular medicinal toxins have been constructed using the principle, where a smaller ‘staple’ SNARE peptide was modelled on the syntaxin SNARE peptide domain. One such new functional protein was a reassembled functional botulinum neurotoxin type A, commonly known as BOTOX [133] and the other was a retargeted clostridial neurotoxin, which utilized a botulinum type A protease/translocation unit from Clostridium botulinum (devoid of the motor-neuron-targeting domain) and the tetanus toxin binding domain from Clostridium tetani (normally capable to target central neurons) [134]. Such a molecular approach to re-engineering functional proteins has achieved not only a safe production of this popular neuronal protease, but also allowed the complete re-engineering of the molecular targeting, with the systemic toxicity of the original protease being abolished entirely [135]. The same approach was used subsequently for the selective targeting of neuroendocrine cells [31] and the development of nonparalytic botulinum molecules for the targeted control of pain [136]. SNARE peptide-based self-assembly system has proven itself as a convenient and safe tool for neuroscience research, with a plethora of potential medical applications.

**Figure 6:**
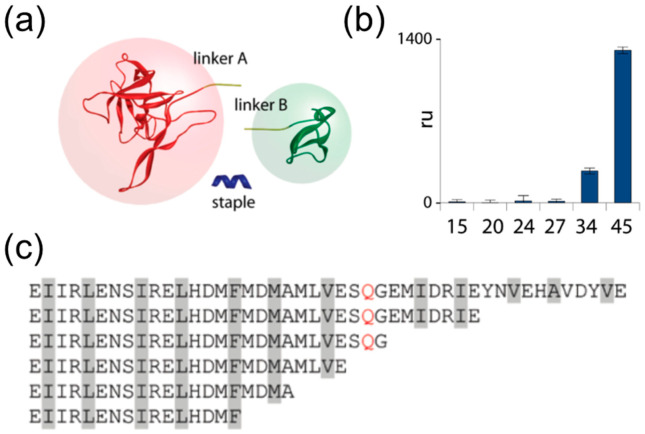
SNARE-inspired ‘stapling’ of proteins together and the effect of the ‘staple’ peptide length on the stability of the assembled complex. Both linkers and the ‘staple’ are based on native SNARE motif polypeptides. The ‘staple’ is a synthetic peptide based on syntaxin sequence. (**a**) The overall scheme explaining the experiment where two large proteins were connected using a short synthetic peptide. The fully assembled complex is stable in 1% SDS solution. (**b**) Stability of stapling by the indicated peptides under denaturing conditions. Bars indicate the amount in resonance units (measured with SPR Biacore technology) of linker B remaining after exposure of the complex to 1% SDS. (**c**) Different length ‘staple’ peptides used; all sequences are based on the SNARE motif of native syntaxin protein. Reproduced from [137] under the ACS AuthorChoice/Editors’ Choice usage agreement.

The use of a single alpha-helical SNARE peptide as a staple provides the additional advantage of relatively easy manipulation of the strength of the protein complex assembled, where alterations of the peptide-staple length could be used to adjust the temperature and stability of the connection and its resistance to denaturants. For example, changing the length of syntaxin-based SNARE peptide yielded protein complexes with varying assembly efficiency and stability against detergents (Figure 6) [137].

**Figure 7:**
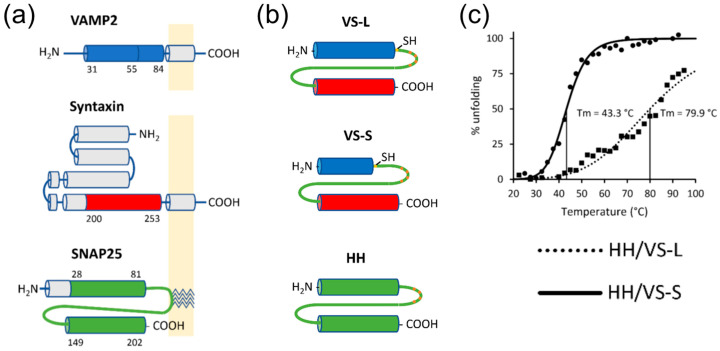
SNARE-inspired re-engineered binary PPI interface for on-demand assembly and controlled disassembly of proteins. (**a**) Schematic of the native SNARE proteins and their transmembrane topology. Color code: domain V from VAMP2 (synaptobrevin protein) is shown in blue, domain S from syntaxin is shown in red, linker and the two SNARE domains from SNAP-25 are shown in green. Cylinders represent α-helixes; the yellow rectangle represents the membrane into which the native SNARE proteins are embedded via an α-helical transmembrane domain (VAMP2 and syntaxin) or palmitoylated cysteine residues (4 zig-zag segments on the SNAP-25). (**b**) Schematic of the re-engineered SNARE mimetics. Cylinders represent SNARE domains highlighted in panel A, whereas the green lines correspond to the naturally occurring linker between the two α-helices of SNAP-25. (**c**) Stability and temperature-dependent disassembly of the SNARE-inspired re-engineered binary PPI complex measured using far-UV synchrotron radiation circular dichroism (SRCD). The range of temperature in which control of protein disassembly is possible extends from Tm = 43 °C to Tm = 80 °C. All panels are reproduced with permission from [138].

The unique features of that assembly are the ability to achieve on-demand non-covalent linking of two tagged recombinant proteins simply by adding a short peptide ‘staple’ to the mixture. The second is that the stability of the complex formed can be adjusted by manipulating the peptide ‘staple’ without changing the two tagged protein being connected. However, the ternary nature of the native-SNARE-based assembly may not provide adequate assembly kinetics. Another substantial development of such a SNARE-inspired polypeptide assembly system was reported in [138], and provides a good example of creative protein re-engineering. Here the sequence specificity and stability of the SNARE coiled coil interface was relied upon and preserved, but the topology of individual SNARE helixes was largely re-engineered to generate a binary PPI system, entirely different from the native SNARE coiled coil assembly (the native SNARE relies upon ternary protein arrangement) (Figure 7). Thus, an engineered binary PPI system allows adjustments to the PPI stability. Varying the length of the VS-L helix between 25 to 54 amino acids varies the temperature stability of the complete assembly from Tm = 43 °C to Tm = 80 °C. The disassembly of the shorter VS-L-containing complex starts within the physiological temperature range, making this system suitable for biomedical applicators, e.g., temperature-controlled therapeutic protein release, as well as for biotechnology and bioengineering applicators, by providing a thermo-responsive biomolecular switch. These and the earlier developments clearly demonstrate the unique exploitable properties of the SNARE molecular self-assembly system beyond its biological role in membrane fusion and exocytosis.

## 5. Conclusions

SNARE proteins govern many forms of intracellular membrane trafficking, a process of critical importance for all eukaryotic cells. In cells, an extensive SNARE interactome organizes, guides, and regulates SNARE proteins to meet specific requirements at the subcellular level. The development of simplified SNARE modulators and SNARE mimetics benefits fundamental studies of cellular function and the practical goals of biomedicine and bioengineering. Presently, functional peptides derived from or modeled on SNARE proteins hold great potential, aided by advances in peptide chemistry, to control structural rigidity and cellular delivery. Peptides derived from the SNARE motif have been shown to regulate (mostly inhibit) endogenous SNARE-complex assembly. The ability of peptides to modify extended binding surfaces in SNARE proteins allows for precise modulation of their function, as exemplified by the identification of stapled peptides that modulate the SNARE complex/calcium sensor primary interface. SNARE proteins have inspired the development of efficient and compact membrane fusogens that take advantage of sequence-specific coiled coil self-assembly mechanism employed by SNARE proteins to drive membrane fusion. Finally, SNARE-derived peptides, which maintain their ability to form highly structured and stable multimolecular complexes show great promise for on-demand protein assembly and the development of stimuli-responsive molecular systems. In all, we anticipate further rapid progress in the engineering of novel functional peptides with a repertoire that expands beyond neuronal SNARE function.

## Supplementary Materials

The following supporting information can be downloaded at: https://www.mdpi.com/article/10.3390/biom12121779/s1, Table S1: Properties of functional peptides modulating SNARE proteins.
