# Supplementary material for: SNARE Modulators and SNARE Mimetic Peptides
Source: Biomolecules. 2022 Nov 29;12(12):1779. doi: 10.3390/biom12121779 (PMC9776023; doi:10.3390/biom12121779)
Supplement: Supplementary file 1 [file biomolecules-12-01779-s001.zip › biomolecules-2033178-supplementary.pdf]

biomolecules

MDPI

SNARE modulators and SNARE mimetic peptides

Mikhail Khvotchev[1,*] and Mikhail Soloviev [2,*]

Supplementary Table S1. Properties of functional peptides modulating SNARE proteins.

No.	Derived from [1]	Membrane fusion [2]	Functional effects	References
1	VAMP (P63045) (1-93)	↓	Inhibited synaptic transmission	[46]
2	VAMP (P63045) (1-96)	↓	Inhibited exocytosis in astrocytes	[47]
3	VAMP (P63045) (1-94)	↓	In reconstituted fusion assays	[48]
4	VAMP (P63045) (25-52)	?	Cytotoxicity, aberrant SNARE complexes	[49]
5	VAMP (P63045) (30-47)	↓	Also, when fused with TAT sequence	[61]
6	VAMP (P63045) (29-56)	↓	In reconstituted fusion assays	[62]
7	VAMP (P63045) (57-92)	↑	In reconstituted fusion assays	[62]
8	SNAP-25 (P60881) (1-180)	↓	Inhibited synaptic transmission	[43]
9	SNAP-25 (P60881) (1-197)	↓	Inhibited synaptic transmission and insulin secretion	[43,44]
10	SNAP-25 (P60881) (187-206)	↓	Inhibited exocytosis in chromaffin cells and synaptic transmission	[51,53]
11	SNAP-25 (P60881) (170-189)	↓	Inhibited synaptic transmission	[53]
12	SNAP-25 (P60881) (181-206)	↓	Inhibited exocytosis in chromaffin cells and synaptic transmission	[52,53]
13	SNAP-25 (P60881) (22-44)	↓	Inhibited exocytosis in chromaffin cells and synaptic transmission	[54]
14	SNAP-25 (P60881) (12-17)	↓	Acetylated, inhibited exocytosis in chromaffin cells	[55]
15	SNAP-25 (P60881) (37-53)	↓	Two staples added; also, when fused with TAT sequence, in reconstituted fusion assays, inhibited mucin secretion	[81,82]
16	Syntaxin 1A (P32851) (1-265)	↓	In reconstituted fusion assays	[48]
17	Syntaxin 1A (P32851) (201-245)	?	Cytotoxicity, aberrant SNARE complexes	[49]
18	Syntaxin 1A (P32851) (229-251)	↓	Inhibited insulin secretion	[56]
19	Syntaxin 1A (P32851) (197-219)	↓	Inhibited insulin secretion	[56]
20	Syntaxin 1A (P32851) (233-245)	↓	Myristoylated, inhibited insulin secretion	[57]
21	Syntaxin 1A (P32851) (200-212)	↓	Myristoylated, inhibited insulin secretion	[57]
22	Syntaxin 1A (P32851) (162-265)	↓	Inhibited synaptic transmission	[58]
23	Syntaxin 1A (P32851) (202-265)	↓	Fused with TAT sequence, inhibited insulin secretion and exocytosis in PC12 cells	[59,60]
24	Syntaxin 1A (P32851) (2-16)	↓	Munc18-1/SNARE complex assembly, inhibited synaptic transmission	[70]
25	Discovered by screening of a peptide library	↓	Ac-SAAEAFAKLYAEAFAKG-NH_2, inhibited exocytosis in chromaffin cells and synaptic transmission	[63]

[1] Protein database ID shown for each SNARE protein and the start/end position of the peptide

[2] Arrows pointing down indicate inhibition of membrane fusion. Question marks indicate that no definitive answer was obtained for the effect on membrane fusion, but aberrant SNARE complexes were detected
